# Supplementary material for: Herpes zoster in older adults in Ontario, 2002–2016: Investigating incidence and exploring equity
Source: PLoS One. 2021 Feb 11;16(2):e0246086. doi: 10.1371/journal.pone.0246086 (PMC7877748; doi:10.1371/journal.pone.0246086)
Supplement: S1 File — (DOCX) [file pone.0246086.s001.docx]

**S1 File –Post Herpetic Neuralgia (PHN) Definition**

We included anyone with a zoster diagnosis in any setting and had 365 days of follow-up following diagnosis (94.4% of the cohort). PHN was flagged for anyone with:

1. Post-zoster neuralgia, nonspecific neuralgia or neuropathic pain code, 90-365 days post zoster
2. Zoster code and prescription consistent with PHN (anticonvulsants, tricyclic antidepressants, capsaicin cream, lidocaine patch) within five days of encounter for zoster, 90-365 days post zoster
3. New anticonvulsant or capsaicin cream or lidocaine patch prescription, 90-180 days post

zoster, excluding those with any of these prescriptions in in the year before initial zoster diagnosis

1. New tricyclic antidepressant 90-180 days post zoster, excluding those with any of the tricyclic antidepressants in the year before initial zoster diagnosis
2. New strong painkiller (i.e., opioid-containing) 90-180 days following zoster with evidence of the drug being prescribed for zoster or PHN previously (within 0-89 days following zoster)
