# Supplementary material for: Herpes zoster in older adults in Ontario, 2002–2016: Investigating incidence and exploring equity
Source: PLoS One. 2021 Feb 11;16(2):e0246086. doi: 10.1371/journal.pone.0246086 (PMC7877748; doi:10.1371/journal.pone.0246086)
Supplement: S1 Table — (DOCX) [file pone.0246086.s002.docx]

**S1 Table.** Average annual population in Ontario aged ≥65 years by characteristic, 2002-2016

|  | **Average annual population, N** |
| --- | --- |
| **Overall** | 1,834,168 |
| **Sex** |  |
| Female | 1,018,279 |
| Male | 815,889 |
| **Age group** |  |
| 65-69 years | 547,113 |
| 70-79 years | 782,739 |
| ≥80 years | 504,316 |
| **Income quintile^a^** |  |
| Income quintile 1 (lowest) | 361,863 |
| Income quintile 2 | 382,641 |
| Income quintile 3 | 360,541 |
| Income quintile 4 | 355,198 |
| Income quintile 5 (highest) | 368,563 |
| **Frail** |  |
| Yes | 205,152 |
| No | 1,629,016 |
| **Age group and frailty** |  |
| 65-69 years |  |
| Frail | 19,358 |
| Not frail | 527,755 |
| 70-79 years |  |
| Frail | 63,702 |
| Not frail | 719,037 |
| ≥80 years |  |
| Frail | 122,092 |
| Not frail | 382,224 |
| **Immunocompromised** |  |
| Yes | 165,529 |
| No | 1,668,639 |

^a^Numbers may not sum to total due to missing data on income quintile (n=5,362 overall)
